# Case Report: Early diagnosis of X-linked Alport syndrome in a pediatric patient and literature review

**DOI:** 10.3389/fped.2026.1786590

**Published:** 2026-05-21

**Authors:** Yushan Gong, Hui Guo, Zhouhang Yang

**Affiliations:** Department of Pediatrics, West China Second University Hospital, Sichuan University, Chengdu, Sichuan Province, China

**Keywords:** Alport syndrome, case report, *COL4A5* mutation, gene mutation, progressive kidney disease

## Abstract

**Background:**

Alport syndrome (AS), one of the most common hereditary kidney diseases, is caused by mutations in genes encoding the α3–α5 chains of type IV collagen, and is characterized by persistent hematuria with or without proteinuria, sensorineural hearing loss, and ocular abnormalities. Progressive renal injury eventually leads to end-stage kidney disease (ESKD). Early interventions can dramatically delay the progression to ESKD. However, AS is often misdiagnosed, leading to mistreatments.

**Case presentation:**

The patient was a 4-year-2-month-old girl, who was incidentally noticed to have foamy morning urine, prompting admission to a local hospital. Repeated urinalyses detected persistent microscopic hematuria in 20 days. Then she was referred to our hospital. Given that her mother also had microscopic hematuria and her maternal grandmother had developed end-stage renal disease of unknown etiology, we excluded infection, autoimmune disorders, and other acquired causes of hematuria, and proceeded with renal biopsy and genetic testing. Electron microscopy showed focal thinning (< 150 nm) and occasional lamellation of the glomerular basement membrane, Light microscopy revealed only mild, focal mesangial proliferation. Trio whole-exome sequencing identified a reported heterozygous missense variant in *COL4A5* (c.1633G > A, p.Gly545Ser) that was inherited from the mother. The same variant was subsequently detected in the affected maternal grandmother. This change is classified as likely pathogenic according to ACMG criteria. Currently, the patient is being treated with an angiotensin-converting enzyme inhibitor (ACEI).

**Conclusion:**

Early renal biopsy and comprehensive genetic analysis in a child with a positive family history of renal disease allowed the expedited diagnosis of X-linked Alport syndrome (XLAS), after the detection of persistent microscopic hematuria in 20 days. Our report highlights that females heterozygous for the *COL4A5* (c.1633G > A, p.Gly545Ser) variant are not merely asymptomatic carriers but may develop renal failure, as illustrated by the disease progression in the maternal grandmother in this pedigree. The single gene *COL4A5* variant, c.1633G > A, in AS patients has been previously reported, and its identification expands the mutational spectrum associated with XLAS and may facilitate future prenatal diagnosis and early therapeutic intervention.

## Background

Alport syndrome (AS), known as hereditary nephritis or oculo-auriculo-renal syndrome, is one of the most common inherited renal disorders. It is caused by mutations in genes encoding the α3, α4 and α5 chains of type IV collagen and is characterized by persistent hematuria with or without proteinuria, sensorineural hearing loss and ocular abnormalities. Progressive renal injury eventually leads to end-stage kidney disease (ESKD). The genetic spectrum of AS includes X-linked, autosomal-recessive, autosomal-dominant and digenic inheritance; the responsible genes are *COL4A3*, *COL4A4* and *COL4A5* (1). These genes encode the α3, α4 and α5 chains that assemble into the α3α4α5 heterotrimer, the principal component of glomerular, cochlear and ocular basement membranes, thereby explaining the renal, auditory and retinal manifestations of the disease (2). According to the mutated gene, AS is classified as XLAS (X-linked AS), ARAS (autosomal-recessive AS), ADAS (autosomal-dominant AS) or, more recently, digenic AS (3); XLAS and ARAS carry the highest risk of renal failure (4). Definitive diagnosis currently requires evidence of glomerular hematuria (± proteinuria) together with characteristic renal-biopsy findings or molecular confirmation. Diagnosis based solely on clinical features and family history is often inconclusive (5). Early diagnosis of AS is essential because blockade of the renin–angiotensin–aldosterone system (RAAS), by using an angiotensin-converting enzyme inhibitor (ACEI) or angiotensin Ⅱ receptor antagonist (ARB), etc., delays the onset of renal failure (6). Genetic testing confirms the diagnosis, identifies at-risk relatives and guides surveillance and management (7). Here we report the early diagnosis and treatment of a pediatric AS case, The child presented with isolated microscopic hematuria and was found to harbor a previously reported *COL4A5* variant, c.1633G > A, inherited from her mother. The child is currently on an ACEI treatment.

## Case report

A 4-year-2-month-old girl was admitted to a local hospital after her parents noticed her foamy morning urine. Routine urinalyses during 20 days at the hospital repeatedly detected microscopic hematuria. The girl was then referred to our hospital. Physical examination was unremarkable. She had no previous illnesses, and her somatic, intellectual, and motor development were all normal. There was no parental consanguinity. Her mother (30 years old) was found to have persistent microscopic hematuria, and her maternal grandmother was on maintenance dialysis for end-stage renal disease of unknown cause; and her father was healthy with no renal disease history.

Admission urinalysis showed 2–4 red blood cells per high-power (HP) field; phase-contrast microscopy revealed 65% dysmorphic erythrocytes, including acanthocytes, microcytes, and ring-shaped cells. The 24-hour urine protein of the child was 0.057 g/24 h (within the normal range). Screening tests for humoral immunity, tumour markers, ANCA (antineutrophil cytoplasmic antibodies), TORCH (toxoplasma gondii, other infections, rubella virus, cytomegalovirus, and herpes simplex virus), auto-antibodies, anti-CCP (cyclic citrullinated peptides), tuberculosis-specific T-cells, pre-transfusion serology, renal ultrasonography, fundoscopy, and high-frequency audiometry were all normal. Because of the striking family history, percutaneous renal biopsy and comprehensive genetic analysis were undertaken.

**Figure 1:**
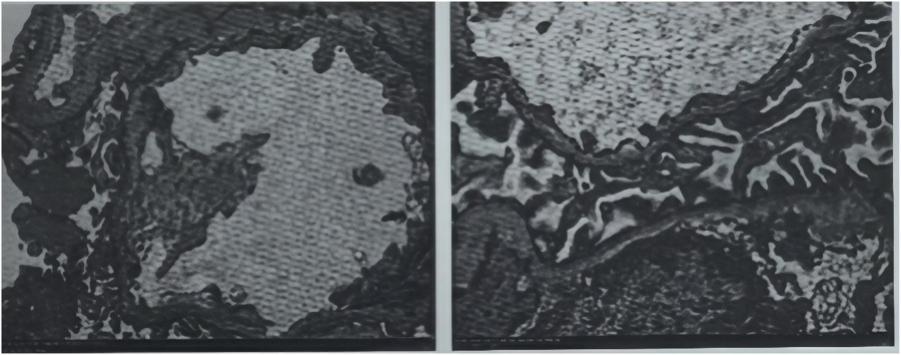
Electron microscopy of the biopsy specimen demonstrated focal thinning of the glomerular basement membrane (< 150 nm) with occasional lamellation.

**Figure 2:**
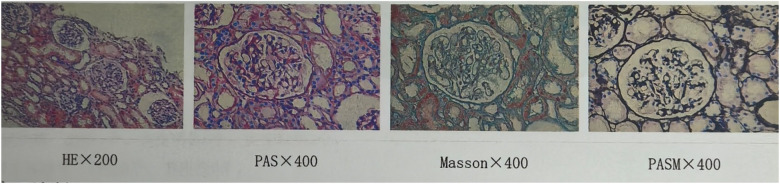
Light microscopy showed only mild, focal mesangial hypercellularity.

Electron microscopy of the biopsy specimen demonstrated focal thinning of the glomerular basement membrane (< 150 nm) with occasional lamellation (Figure 1), whereas light microscopy showed only mild, focal mesangial hypercellularity (Figure 2). Genetic test was performed by trio whole-exome sequencing. Genomic DNA extracted from peripheral blood was enriched by a capture-based protocol and subjected to high-throughput sequencing (KingMed Diagnostics). Reads were aligned to the human reference genome (GRCh37/hg19) using BWA; single-nucleotide variants and small insertions/deletions were called with GATK and annotated with ANNOVAR. Variant filtering and pathogenicity assessment followed the ACMG guidelines, with emphasis on established nephropathy genes. All candidate variants were verified by Sanger sequencing. A heterozygous missense variant in *COL4A5* (NM_033380.2: c.1633G > A, p.Gly545Ser) was identified in the child. This substitution, converting guanine to adenine at nucleotide 1633, results in the replacement of glycine by serine at codon 545. This change is classified as likely pathogenic according to ACMG (The American College of Medical Genetics and Genomics) criteria. The child was diagnosed with X-linked AS, and is currently treated with enalapril maleate tablets (an ACEI) in the outpatient clinic of our hospital. Because she was also suffering from vitamin D deficiency, she has been also supplemented with vitamin D. After AS diagnosis and treatment, the child was followed up every month for routine urinalysis, which revealed 2+ to 3 +red blood cells/HP and negative urine protein. The patient does not have anemia; her routine blood examination showed a hemoglobin level of 119 g/L (reference range: 110–140 g/L), which is within the normal range. The patient currently has no other symptoms.

**Figure 3:**
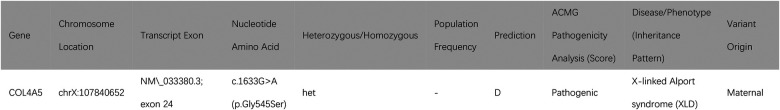
Result of genetic test indicated that the girl's heterozygous variant in *COL4A5* was inherited from her mother.

**Figure 4:**
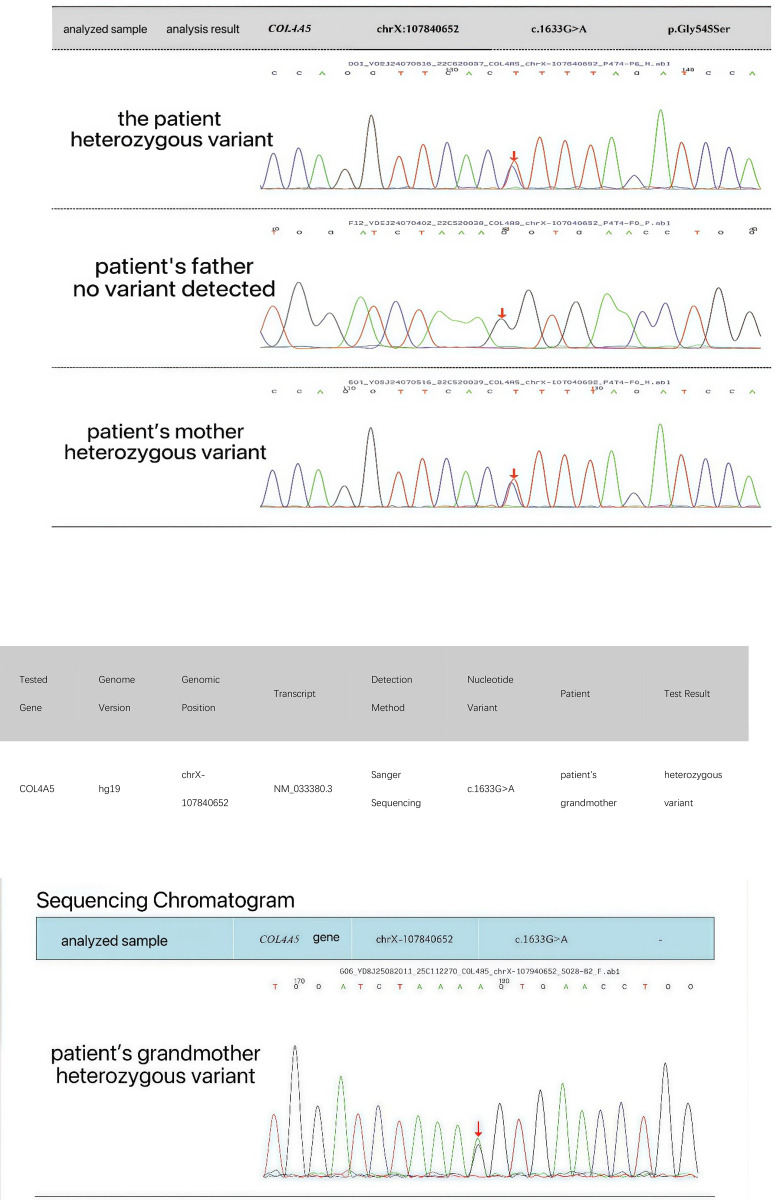
Sanger sequencing results of the proband, her parents, and grandmother. Her mother and maternal grandmother carried the same heterozygous variant, whereas her father did not.

Urinalysis for her mother (30-year-old) confirmed the persistence of microscopic hematuria and negative proteinuria. Renal function analysis showed that her creatinine level and eGFR (estimated glomerular filtration rate) were within the normal ranges. At present, the mother's hearing and vision were normal, and her blood pressure was within the normal range. Fundus screening was not done, and the mother has not received any special treatment so far. Genetic test showed that the mother carried the same heterozygous variant as the girl, whereas the father did not (Figures 3, 4). The child's maternal grandmother started to have abnormal renal function in her 30s. At the age of 56 years, she was found to have renal failure after the presence of oliguria and edema. Recently, she has been undergoing renal dialysis treatment. The same heterozygous mutation was confirmed in the affected maternal grandmother (Figure 4).

## Discussion and conclusions

Alport syndrome (AS) usually becomes apparent during childhood. Early interventions can dramatically delay the disease progression to ESKD (1). However, AS is often misdiagnosed, leading to mistreatments. Therefore, early diagnosis and treatment are critical. Here we report the early diagnosis and treatment of a pediatric female patient with AS. The 4-year-2-month-old girl was referred to our hospital for further examination following the identification of persistent microscopic hematuria at a local hospital in 20 days. The initial visit to the local hospital was motivated by the parents' concern about foamy morning urine. A strongly positive family history of renal disease prompted us to perform timely renal biopsy and comprehensive genetic testing for the girl, leading to an expedited definitive diagnosis of XLAS.

AS results from defects in the type IV collagen network, the principal structural component of glomerular, cochlear and ocular basement membranes. Six genetically distinct α-chains (α1-α6), encoded by *COL4A1-COL4A6*, have been identified. Approximately 85% of AS cases arise from pathogenic variants in *COL4A5* (or contiguous deletions involving *COL4A5* and *COL4A6*) and follow X-linked dominant inheritance, whereas ∼15% are attributable to bi-allelic or, less often, mono-allelic mutations in *COL4A3* or *COL4A4* and are inherited in an autosomal fashion. XLAS is the commonest genotypic class, accounting for about 80% of all patients (8).

Clinically, over 90% of individuals with *COL4A5* variants develop persistent microscopic hematuria and more than 60% develop proteinuria. Long-term studies showed that 15%–30% of women with a heterozygous *COL4A5* variant develop kidney failure by age of 60 years, whereas approximately 90% of affected males require renal-replacement therapy before age of 40 years (9). Co-existing heterozygous pathogenic variants in *COL4A3* or *COL4A4* may exacerbate the phenotype by further destabilizing the α3α4α5 network, thereby accelerating glomerular basement-membrane deterioration (10). Sensorineural hearing loss (SNHL) is observed in 32%–83% of XLAS males but in only 6%–28% of females, and males also become symptomatic earlier (11). Ocular abnormalities—anterior lenticonus and perimacular fleck retinopathy—usually appear after renal impairment has developed and are rarely encountered before adolescence.

The girl with XLAS reported here had neither proteinuria nor audiologic or ophthalmologic involvement, except persistent microscopic hematuria, on admission. Whole-exome sequencing for the patient identified a heterozygous missense variant in *COL4A5* (c.1633G > A, p.Gly545Ser). Her condition is currently managed in the outpatient clinic with an ACEI for delaying the progression of the disease. The girl's genetic change was inherited from her mother. The 30-year-old mother was found to have persistent microscopic hematuria without proteinuria, and she opted not to receive any special treatment. The same variant was subsequently detected in the affected maternal grandmother, who did not pay attention to the problem of abnormal renal function in the early stage, but was found to have renal failure at the age of 56 years, and is currently on renal dialysis.

The genetic variation, *COL4A5* (c.1633G > A, p.Gly545Ser) found in this family, has been previously reported. In a recently published case report of AS, a family with digenic variation in *COL4A5* (c.1633G > A) and *NPHS1* (c.2417C > A) was described. A 5-year-old girl, her father and 3-year-old sister carried the same variation combination. The child showed recurrent gross hematuria and proteinuria, without hearing and eye abnormalities, and showed highly abnormal capillary and mesangial malformations under the electron microscope of renal biopsy (12).

XLAS can manifest in infancy, and isolated microscopic hematuria is often misattributed to nutcracker syndrome, acute post-infectious glomerulonephritis, or IgA nephropathy. Because renal biopsy is frequently deferred for ≥ 6 months in children with isolated hematuria, the correct diagnosis may be delayed. Now, it is believed that early detection and early treatment of pediatric AS patients may delay the disease progression to renal failure or even prevent from developing renal failure in the future. We therefore recommend that unexplained glomerular hematuria in a child should prompt urinalysis and renal-function assessment in all first-degree relatives; if the family history screen is positive, early renal biopsy and targeted genetic testing should be performed to distinguish hereditary from acquired glomerular diseases and to avoid inappropriate therapy.

This case report adds valuable information to the diagnosis and treatment of AS. The identification of the previously reported single gene *COL4A5* variant, c.1633G > A, in AS patients expands the mutational spectrum associated with XLAS. The finding may be beneficial for prenatal diagnosis in the future. Early intervention after early diagnosis of AS can be carried out in the patients carrying the pathogenic variation, so as to delay the progression to ESKD.

## Data Availability

The original contributions presented in the study are included in the article/Supplementary Material, further inquiries can be directed to the corresponding authors.
